# Alteration of m6A RNA Methylation in Heart Failure With Preserved Ejection Fraction

**DOI:** 10.3389/fcvm.2021.647806

**Published:** 2021-03-05

**Authors:** Beijian Zhang, Yamei Xu, Xiaotong Cui, Hao Jiang, Wei Luo, Xinyu Weng, Yun Wang, Yuhong Zhao, Aijun Sun, Junbo Ge

**Affiliations:** ^1^Department of Cardiology, Zhongshan Hospital, Fudan University, Shanghai Institute of Cardiovascular Diseases, Shanghai, China; ^2^Key Laboratory of Viral Heart Diseases, National Health Commission, Shanghai, China; ^3^Key Laboratory of Viral Heart Diseases, Chinese Academy of Medical Sciences, Shanghai, China; ^4^Tianshan Hospital of Traditional Chinese Medicine, Shanghai, China; ^5^Institutes of Biomedical Sciences, Fudan University, Shanghai, China

**Keywords:** heart failure with preserved ejection fraction, N6-methyladenosine, epitranscriptomics, METTL3, FTO

## Abstract

**Background:** Heart failure with preserved ejection fraction (HFpEF) is a heterogeneous disease, in which its pathogenesis is very complex and far from defined. Here, we explored the N^6^-methyladenosine (m6A) RNA methylation alteration in patients with HFpEF and mouse model of HFpEF.

**Methods:** In this case–control study, peripheral blood mononuclear cells (PBMCs) were separated from peripheral blood samples obtained from 16 HFpEF patients and 24 healthy controls. The change of m6A regulators was detected by quantitative real-time PCR (RT-PCR). A “two-hit” mouse model of HFpEF was induced by a high-fat diet and drinking water with 0.5 g/L of *N*^ω^-nitro-l-arginine methyl ester (L-NAME). MeRIP-seq was used to map transcriptome-wide m6A in control mice and HFpEF mice, and the gene expression was high-throughput detected by RNA-seq.

**Results:** The expression of m6A writers *METTL3, METTL4*, and *KIAA1429*; m6A eraser *FTO*; and reader *YTHDF2* was up-regulated in HFpEF patients, compared with health controls. Furthermore, the expression of *FTO* was also elevated in HFpEF mice. A total of 661 m6A peaks were significantly changed by MeRIP-seq. Gene Ontology (GO) analysis revealed that protein folding, ubiquitin-dependent ERAD pathway, and positive regulation of RNA polymerase II were the three most significantly altered biological processes in HFpEF. The pathways including proteasome, protein processing in the endoplasmic reticulum, and PI3K-Akt signaling pathway were significantly changed in HFpEF by Kyoto Encyclopedia of Genes and Genomes (KEGG) pathway analysis.

**Conclusions:** The expression pattern of m6A regulators and m6A landscape is changed in HFpEF. This uncovers a new transcription-independent mechanism of translation regulation. Therefore, our data suggest that the modulation of epitranscriptomic processes, such as m6A methylation, might be an interesting target for therapeutic interventions.

## Introduction

Heart failure is the main cause of mortality worldwide. Furthermore, heart failure with preserved ejection fraction (HFpEF) accounts for 50% or higher of heart failure. With the development of aging and the increasing prevalence of obesity, hypertension, and diabetes, this ratio will be higher (1). Because of the complex pathophysiological mechanism and heterogeneity of this syndrome, there is no evidence-based therapy for HFpEF, and treatment proven effective in heart failure with reduced ejection fraction (HFrEF) cannot improve survival in HFpEF (2). In this setting, exploration of various molecular and cellular mechanisms contributing to the morbidity of HFpEF is very crucial.

Recently, emerging evidences have demonstrated that epigenetics plays critical roles in the pathophysiological responses of HFpEF, such as DNA methylation, chromatin remodeling, histone modifications, and microRNA-depending gene expression (3). DNA methylation has been shown a causality role in diabetes-induced HFpEF (4). Aging affects the progress of HFpEF through the regulation of DNA methylation and histone modifications (5). Moreover, the alteration of microRNAs, such as down-regulation of miRNA-1 and up-regulation of miRNA-195, controls cardiac hypertrophy, oxidative stress, ischemic susceptibility, and fibrosis in HFpEF through histone modification (3, 6). Recently, Wallner et al. (7) reported that inhibition of histone deacetylases (HDAC) activity with suberoylanilide hydroxamic acid improves cardiopulmonary function, i.e., preserved lung structure, compliance, blood oxygenation, and reduced perivascular fluid cuffs around extra-alveolar vessels in HFpEF. Furthermore, Jeong et al. (8) found that HDAC inhibition with ITF2357 (givinostat) ameliorates the impairment of cardiac myofibril relaxation, cardiac fibrosis, and cardiac hypertrophy and changes in cardiac titin and myosin isoform expression in Dahl salt-sensitive rats with HFpEF, indicating that epigenetic regulation also significantly contributes to HFpEF.

N^6^-methyladenosine (m6A) is the most common post-transcriptional modification of mRNA in mammals (9, 10). Recent studies have demonstrated that it is important for the regulation of various biological processes, such as embryonic development, cell differentiation, regeneration, and tumorigenesis (11–15). However, a study related to m6A in the cardiovascular field is still rare. It is reported that the global level of m6A is increased in myocardial infarction, ischemia–reperfusion injury, and HFrEF, and decreased m6A may enhance autophagic flux and improve cardiac function (16–19). Consistent with these roles, m6A modification is emerging as a key pathway influencing the pathological progress of HFrEF. However, how m6A modification affects heart function and which underlying mechanisms mediate these changes remain unknown. Given the critical role of m6A in regulating mRNA modification related to various biological processes by influencing mRNA stability, splicing, translation, and localization (20–26), it is reasonable to speculate that m6A may be involved in HFpEF. However, its role in HFpEF has not been studied.

Lacking relevant experimental models to accurately recapitulate the heterogeneity of this complex disease leads to the lack of effective treatments for HFpEF, as it is increasingly recognized as a complex interaction of multiple impairments throughout the body rather than cardiomyocyte disorder (27). Multiple comorbidities, such as diabetes, obesity, and hypertension, have been demonstrated to increase the risk of HFpEF (28). Recently, Hill et al. (29) proposed a “two-hit” mouse model of HFpEF, which mimicked concomitant metabolic and hypertensive stress in mice. In this model, a high-fat diet (HFD) induces the metabolic stress (obesity, glucose intolerant, and metabolic syndrome), and hypertension is caused by a drug called *N*^ω^-nitro-l-arginine methyl ester (L-NAME), which inhibits nitric oxide (NO) synthase. This model recapitulates the numerous systemic and cardiovascular characteristics of HFpEF, including impaired cardiac filling, cardiac hypertrophy, cardiac fibrosis, reduced myocardial capillary density, pulmonary hyperemia, reduced exercise tolerance, and increased levels of inflammatory markers (29). Thus, this *bona fide* model of HFpEF was used in this study.

In order to explore the epigenetic modifications of RNA in HFpEF and their diagnostic value, we analyzed the m6A regulators in patients with HFpEF and healthy controls and the m6A methylation profiles in the setting of a “two-hit” mouse model of HFpEF (29). By analyzing of RNA and m6A methylation levels, we have identified potential novel targets that can provide a basis for further intervention in HFpEF.

## Methods

### Patients and Control Subjects

In the part of case–control study, 16 HFpEF patients in our hospital from November 2020 to December 2020 were enrolled, and 24 cases who took health examination at the same period were recruited as healthy controls. The study complied with the Declaration of Helsinki and was registered (ChiCTR2000040038). The research program was approved by the ethics committee (No. B2020-356R) at Zhongshan Hospital, Fudan University, China. All patients provided written informed consent. Patients with HFpEF were eligible for the study (30). Exclusion criteria included (1) age <18 years, (2) participate in other clinical trials in the previous 3 months, (3) cancers, (4) chronic kidney disease at stage 2 or above, (5) severe hepatic insufficiency, (6) blood systemic diseases, such as leukemia, and (7) unlikely cooperation in the study. Baseline characteristics of study subjects were obtained, including age, gender, body mass index, hypertension, diabetes, atrial fibrillation, coronary heart disease, laboratory parameters, and echocardiography parameters.

### Blood Sampling and Peripheral Blood Mononuclear Cells Extraction

Peripheral blood samples (8–10 ml) were collected into ETDA anticoagulant vacutainer (Becton Dickinson, San Jose, CA, USA) from HFpEF patients and healthy controls. Peripheral blood mononuclear cells (PBMCs) were extracted by Ficoll-isopaque centrifugation as mentioned previously (31). Briefly, peripheral blood samples were centrifuged at 3,000 rpm for 10 min to obtain complete blood cell. After dilution with phosphate-buffered saline (PBS) at a ratio of 1:1, the diluted complete blood cell was transferred to lymphocyte separation medium (TBDsciences, Tianjin, China) and then centrifuged at 3,000 rpm for 10 min again to obtain PBMCs.

### Animals and a “Two-Hit” HFpEF Model

Eight-week-old, male C57/BL6 mice were purchased from Shanghai Model Organisms Center, Inc. (Shanghai, China). A “two-hit” mouse model of HFpEF was induced as described previously (29). Briefly, HFpEF mice were fed with a HFD [60% kilocalories from fat (lard)] and drinking water with 0.5 g/L of L-NAME (Sigma, N5751) for 10 weeks; control mice were fed with a standard diet. Mice were maintained in a 12/12-h light/dark cycle environment with a 22°C constant temperature. All animal experimental processes followed the Guide for the Care and Use of Laboratory Animals, published by the US National Institutes of Health (NIH publication no. 85-23, revised 1996), and were reviewed and approved by the animal ethics committee at Zhongshan Hospital, Fudan University, China.

### RT-PCR

The total RNA of PBMCs and heart tissue was extracted with TriZol reagent (Invitrogen, USA), and the quality and quantity of RNA were assessed by NanoDrop 2000 (Thermo Fisher Scientific, USA). The reverse transcription was performed by using PrimeScript RT reagent kit (Takara, Japan). Then, real-time PCR (RT-PCR) was performed by using SYBR Premix Ex Taq II (Takara, Japan) in CFX96 Real-Time System (Bio-Rad, USA). Relative gene expression was normalized by 18S. The primers are listed in Supplementary Table 1.

### MeRIP-seq

Total RNA was extracted from heart tissue with TriZol reagent (Invitrogen, USA), and polyA^+^ RNA was enriched from total RNA with oligo-dT magnetic beads. Then, the polyA^+^ RNA was fragmented to ~100 nt long fragments by using RNA fragmentation buffer (Millipore Sigma, USA). The fragment RNA was divided into two parts; one was enriched with m6A antibody that could capture m6A for m6A-IP, and the other was used as input to construct normal transcriptome sequencing library. After the RNA fragment with m6A was enriched, the conventional sequencing library was constructed. The constructed sequencing libraries were sequenced by using the sequencing platform Illumina Hiseq X Ten (OE Biotech, China).

### GO Analysis and KEGG Pathway Analysis

In order to annotate the altered m6A peaks, Gene Ontology (GO) enrichment analysis was used to describe the function of genes related to differential peaks. GO analysis of differentially expressed peaks was performed by using R based on the hypergeometric distribution. The number of genes related to the altered peaks in each GO term was counted, and the significance of enrichment of genes in each GO term was calculated by hypergeometric distribution test. GO categories from “biological process,” “cellular component,” and “molecular function” were extracted and plotted with their –log_10_ *P*-value. Moreover, Kyoto Encyclopedia of Genes and Genomes (KEGG) pathway analysis was performed by using R, and hypergeometric distribution test was used to calculate the significance of genes related to altered peaks in each pathway term.

### Statistical Analysis

All statistical analysis was performed by GraphPad Prism 7.0. Data were expressed as mean ± standard deviation (SD). Normal distribution was evaluated by Shapiro–Wilk test. Differences between two groups were determined by using unpaired Student's *t*-test. Furthermore, the association between m6A regulators and blood fasting glucose and blood lipids was determined by Pearson correlation test. Statistical significance was considered when *P* < 0.05.

## Results

### Expression of m6A Regulators Was Changed in HFpEF Patients and HFpEF Mice

**Figure 1:**
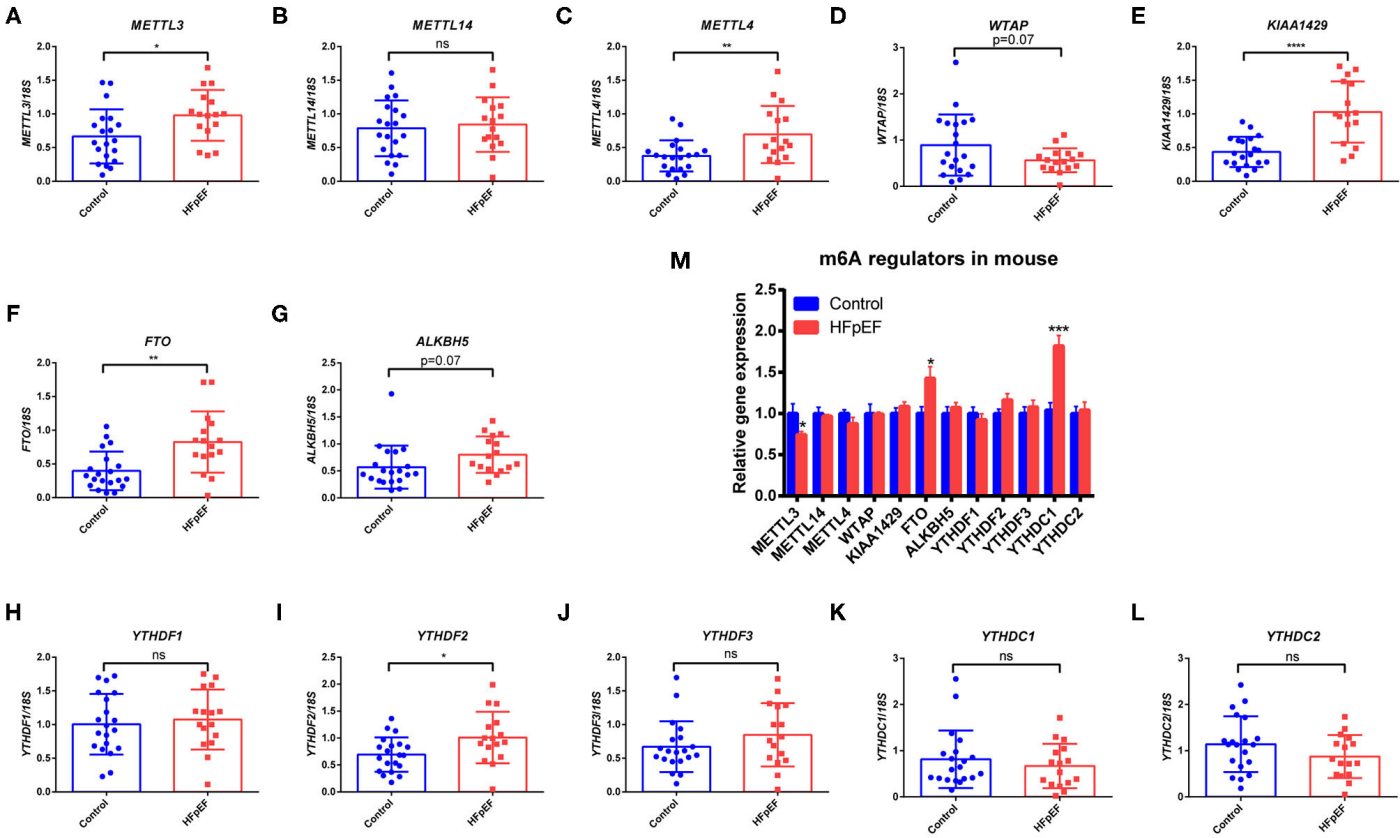
The expression pattern of m6A regulators in HFpEF patients and mice. **(A–L)** RT-PCR of m6A writers (*METTL3, METTL14, METTL4, WTAP*, and *KIAA1429*), erasers (*FTO* and *ALKBH5*), and readers (*YTHDF1-3* and *YTHDC1-2*) in peripheral blood of HFpEF patients (*n* = 16) and healthy controls (*n* = 24). **(M)** RT-PCR of m6A regulators in the hearts in a “two-hit” mouse model of HFpEF (*n* = 6 per group). Data were shown as mean ± SD, and the differences were determined by Student's *t*-test. *P < 0.05, **P < 0.01, ***P < 0.001, and ****P < 0.0001, compared with the control group.

A total of 16 HFpEF patients and 24 healthy controls were analyzed in this study. The average age was 53 ± 15 years, and 65% of them were male. In order to investigate whether m6A methylation status was changed in HFpEF patients, we evaluated the m6A regulators in peripheral blood in HFpEF patients and healthy controls by RT-PCR: writers: *METTL3, METTL14, WTAP, METTL4*, and *KIAA1429*; erasers: *FTO* and *ALKBH5*; and readers: *YTHDF1-3* and *YTHDC1-2*. The expression of *METTL3, METTL4, KIAA1429, FTO*, and *YTHDF2* was significantly up-regulated in HFpEF patients (Figures 1A,C,E,F,I), compared with healthy controls. Furthermore, the expression of *WTAP* has a decreased trend (Figure 1D), and *ALKBH5* has an increased trend (Figure 1G), but the significance was near the border (*P* = 0.07). The expression of *METTL14, YTHDF1, YTHDF3, YTHDC1*, and *YTHDC2* remained unchanged between these two groups (Figures 1B,H,J–L). Then, we detected the m6A regulators in the hearts of HFpEF mice. In addition, we found that *FTO* was also up-regulated in HFpEF mice compared with control mice (Figure 1M), consistent with the finding in peripheral blood of HFpEF patients. However, *METTL3* was down-regulated, and the expression of *METTL4, KIAA1429*, and *YTHDF2* was not significantly changed (Figure 1M). Interesting, the expression of *YTHDC1* was up-regulated in HFpEF mouse (Figure 1M), which remained unchanged in HFpEF patients. The expression pattern changes of these m6A regulators could lead to a dynamic change in the m6A methylation in HFpEF patients and HFpEF mice.

### Association of m6A Regulators With Risk of HFpEF

**Figure 2:**
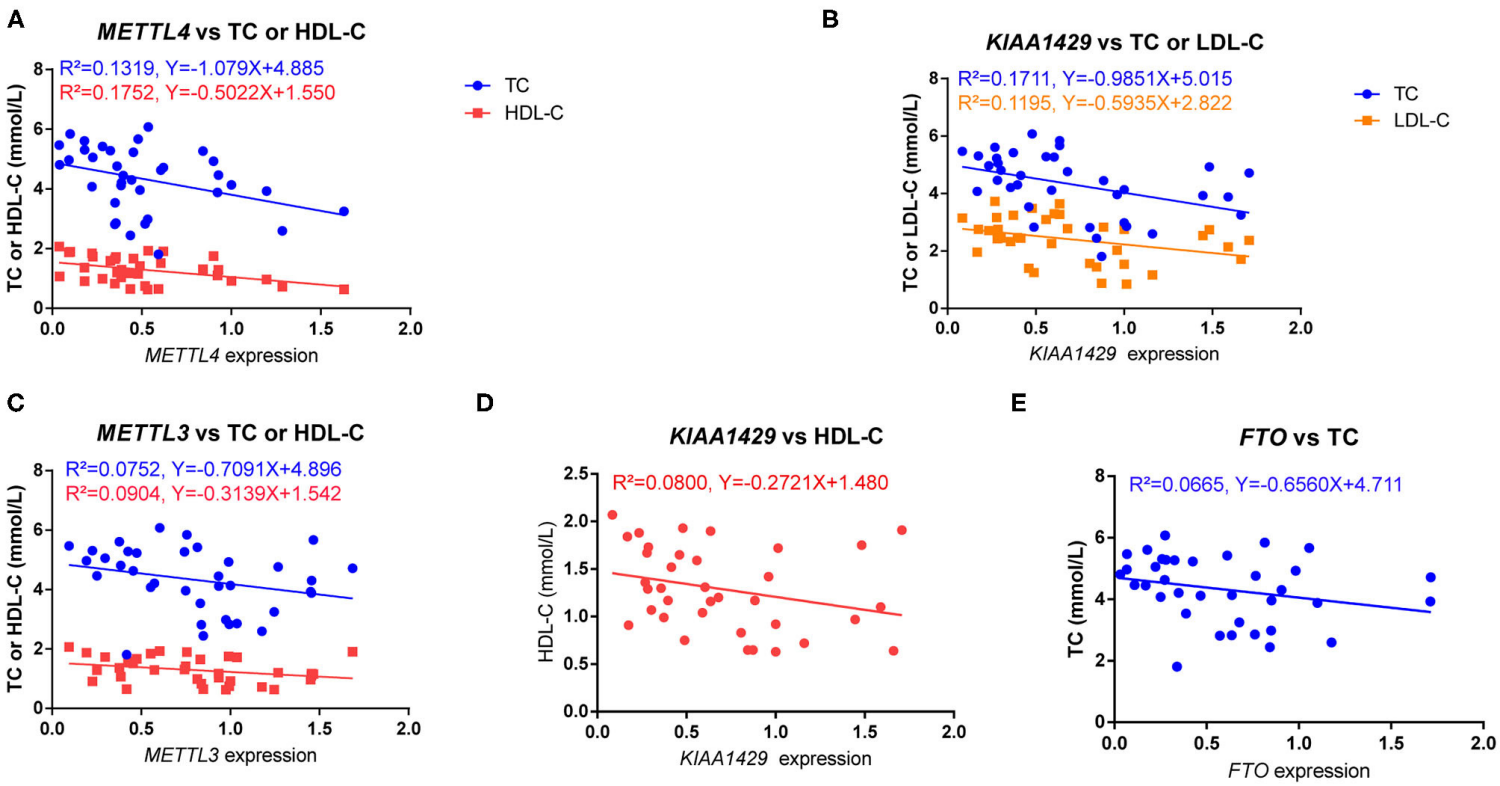
The relationship of m6A regulators with serum lipids. **(A)** The expression of *METTL4* was negatively correlated with TC (*r* = −0.3632, *P* = 0.0295) and HDL-C (*r* = −0.4186, *P* = 0.0111). **(B)** The expression of *KIAA1429* was negatively correlated with TC (*r* = −0.4137, *P* = 0.0121) and LDL-C (*r* = −0.3457, *P* = 0.0389). **(C–E)** The association of *METTL3, KIAA1429*, and *FTO* with TC or HDL-C was near the border of significance (*P*-value range from 0.05 to 0.1). The relation was determined by Pearson correlation test.

It is known that blood lipids and fasting glucose are risk factors for HFpEF (32, 33); then, we explored the association of the m6A regulators with total cholesterol (TC), triglycerides (TG), high-density lipoprotein cholesterol (HDL-C), low-density lipoprotein cholesterol (LDL-C) and fasting glucose of HFpEF patients by Pearson correlation test. Furthermore, we found that the expression of *METTL4* was negatively correlated with TC (r = −0.3632, *P* = 0.0295) and HDL-C (*r* = −0.4186, *P* = 0.0111) (Figure 2A). *KIAA1429* was negatively correlated with TC (*r* = −0.4137, *P* = 0.0121) and LDL-C (*r* = −0.3457, *P* = 0.0389) (Figure 2B). In addition, the association of *METTL3, KIAA1429*, and *FTO* with TC or HDL-C was near the border of significance (*P*-value range from 0.05 to 0.1) (Figures 2C–E). However, there was no correlation of m6A regulators with fasting glucose (data not shown).

### Topological Distribution of m6A Peaks in HFpEF Mice

**Figure 3:**
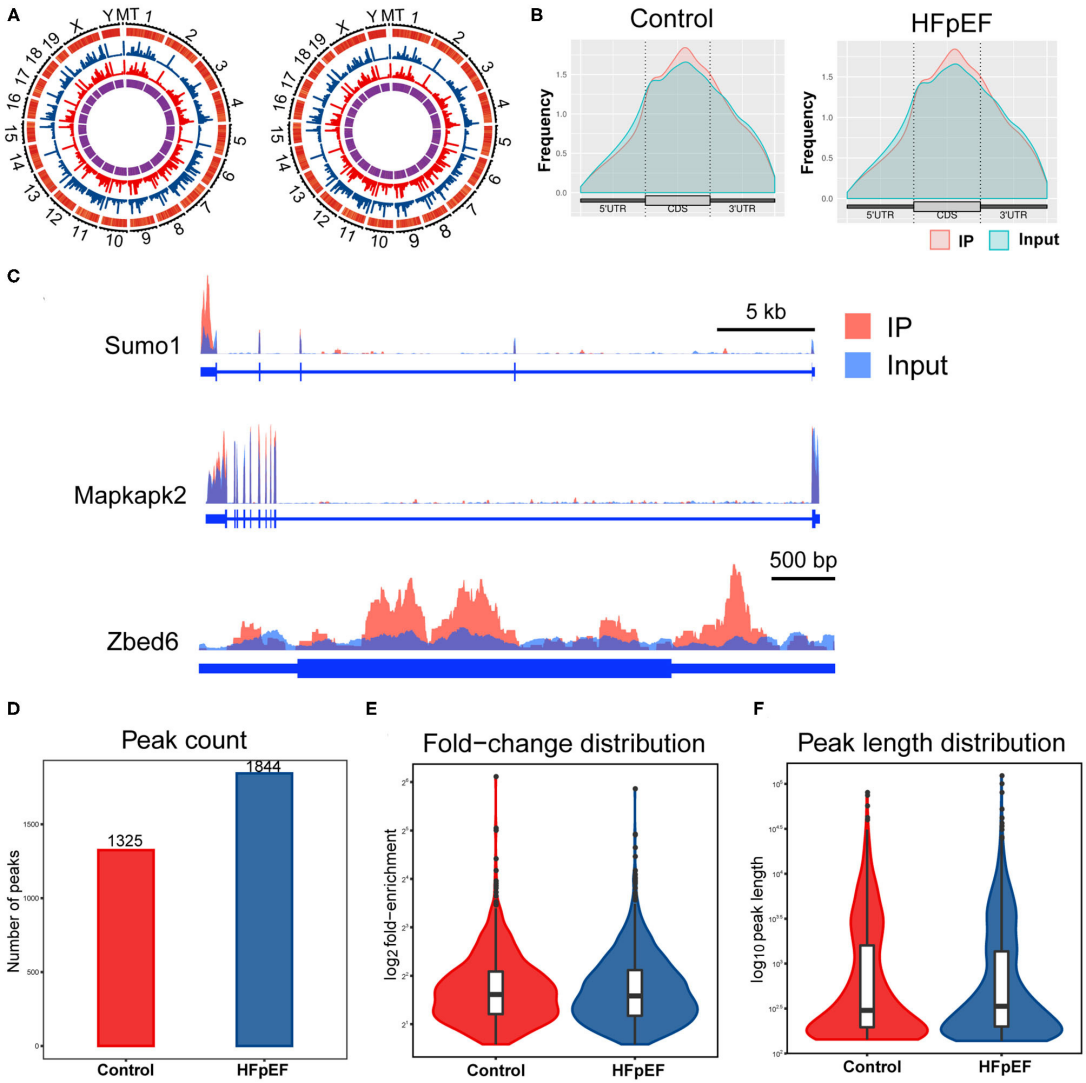
The topological distribution of m6A peaks in HFpEF mice. **(A)** The distribution patterns of m6A peaks in different chromosomes of control mice and HFpEF mice. **(B)** The distribution patterns of m6A methylation peaks in gene structures of mRNA. **(C)** Visualization of representative m6A peaks in the genes of *Sumo1, Mapkapk2*, and *Zbed6*, using Integrative Genomics Viewer. The peak of *Sumo1* was located at the 3′UTR region, the two peaks of *Mapkapk2* were both located at the 3′UTR regions, and the peaks of *Zbed6* were located at the CDS and the 5′UTR regions. **(D)** The number of m6A peaks in the indicated groups. **(E)** The fold-enrichment of indicated groups. **(F)** The peak length distribution of indicated groups.

In order to determine the m6A modification levels of the HFpEF mice and control mice, we performed a transcriptome-wide m6A-seq analysis by MeRIP-seq. Compared with the high-throughput data between IP samples and their corresponding inputs, m6A methylation peaks were distinguished, including 1,852 distinct m6A peaks for 1,182 genes in the HFpEF group and 1,326 m6A peaks for 899 genes in the control group (Supplementary Table 2). The chromosomes with the most m6A modification sites in control mice were chromosomes 2, 4, and 7 with 104, 94, and 87 m6A modification sites in 69, 61, and 66 genes, respectively (Figure 3A, Supplementary Table 2). Furthermore, in HFpEF mice, they were chromosomes 4, 11, and 2 with 130, 125, and 122 m6A methylation sites in 79, 84, and 77 genes, respectively (Figure 3A, Supplementary Table 2). Moreover, the number of m6A methylation sites on the genes ranged from 1 to 13 in both groups, with 86.80% of genes having one or two m6A modification sites and 13.20% of genes having three or more m6A modification sites in the HFpEF group and 89.77% of genes having one or two m6A modification sites and 10.23% of genes having three or more m6A modification sites in the control group (Supplementary Table 2). For example, *Dync1h1* located on chromosome 12 was identified with the maximum number of m6A modification sites (13 sites) in the HFpEF group, and *Cmya5* located on chromosome 13 was also identified with the maximum number of m6A modification sites (13 sites) in control mice (Supplementary Table 2).

Then, the distribution patterns of m6A peaks across mRNA transcripts were analyzed, and we found that the frequency of m6A peaks across all transcripts was mostly distributed on the coding sequence (CDS) region and there was also distinct enrichment at the 5′UTR and 3′UTR regions (Figure 3B). Three representative genes (*Sumo1, Mapkapk2*, and *Zbed6*) were chosen to present the m6A modification pattern (Figure 3C). The peak of *Sumo1* was located at the 3′UTR region, the two peaks of *Mapkapk2* were both located at the 3′UTR regions, and the peaks of *Zbed6* were located at the CDS and the 5′UTR regions. A total of 1,325 and 1,844 peaks were identified in control mice and HFpEF mice (Figure 3D), respectively. The average logarithmic fold-enrichment in HFpEF mice and control mice was 3.72 (Figure 3E). Furthermore, the average peak length of HFpEF mice was 1,799.86 bp and 1,943.19 bp in control mice (Figure 3F).

### Significant m6A Methylation Alteration in HFpEF

**Figure 4:**
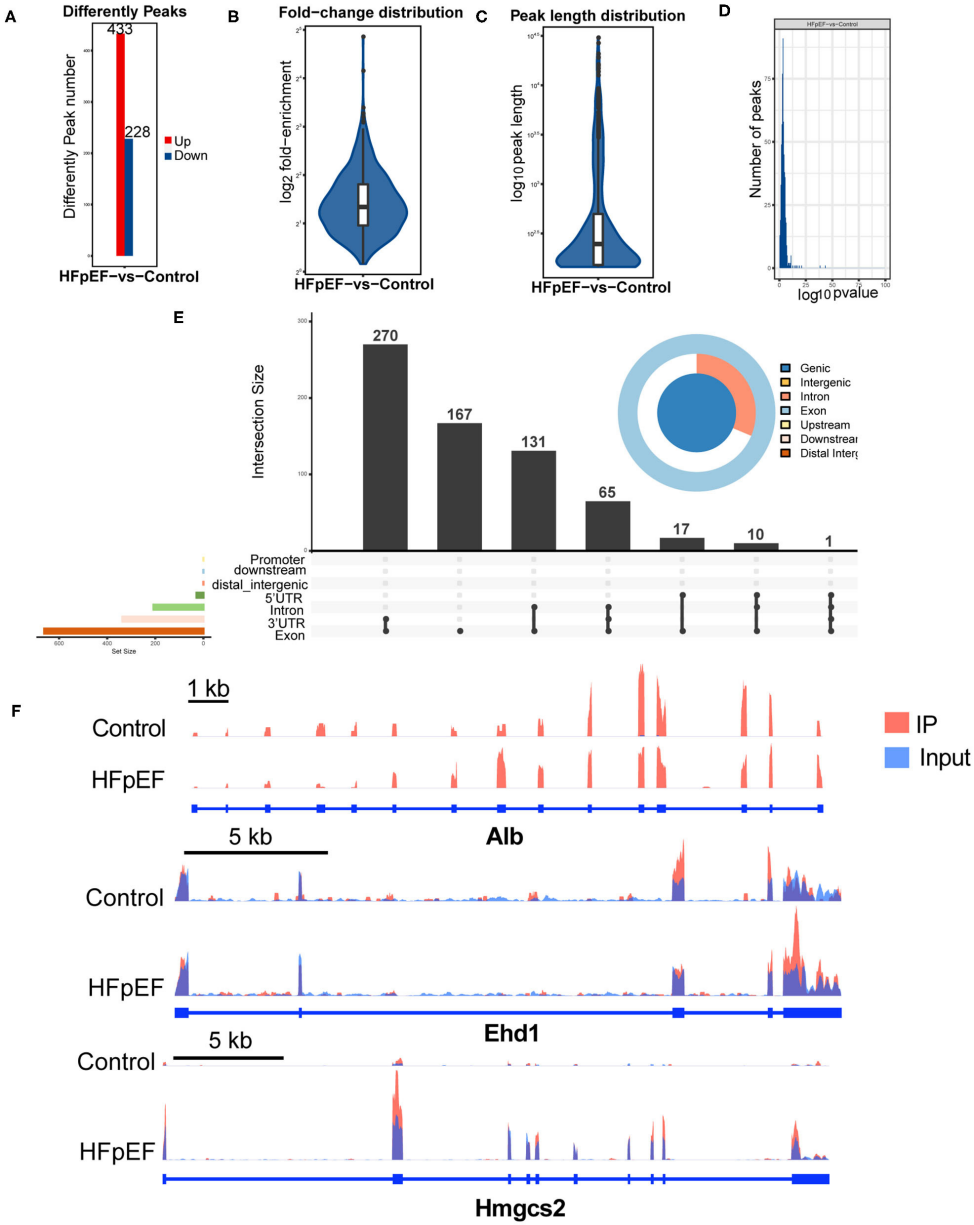
The difference analysis of identified m6A peaks in HFpEF mice and control mice. **(A)** The up-regulated and down-regulated peak numbers in HFpEF mice, compared with control mice. **(B–D)** The fold-enrichment **(B)**, length **(C)**, and distribution of *P*-value **(D)** of altered m6A peaks. **(E)** The exact distribution pattern of significantly altered peaks in HFpEF mice. There were 270 peaks distributed in the 3′UTR and exon regions, 167 peaks in the exon region, and 131 peaks in the intron and exon regions. **(F)** Three representative genes with significantly changed peaks. The m6A levels of *Alb, Ehd1*, and *Hmgcs2* were significantly up-regulated in HFpEF by 29, 8.34, and 3.27 times, respectively.

To clarify the function of m6A modification in HFpEF, we compared the m6A modification levels of mouse hearts between the HFpEF mice and control mice. A total of 661 m6A peaks were significantly altered between two groups, and 443 of them were up-regulated, and 228 peaks were down-regulated in the HFpEF group (Figure 4A), compared with the control group. The top 20 differently expressed m6A marked mRNAs were presented in Table 1. Compared with the input sample, the average logarithmic fold-enrichment of differently expressed peaks was 2.98 (Figure 4B), and the average peak length was 1,140.81 bp (Figure 4C). The distribution of *P*-value of the altered peaks was presented in Figure 4D. The exact distribution pattern of altered peaks in HFpEF mice was shown in Figure 4E. There were 270 peaks distributed in the 3′UTR and exon regions, 167 peaks in the exon region, and 131 peaks in the intron and exon regions. Three representative mRNAs with significantly altered peaks were shown in Figure 4F. The m6A methylation levels of *Alb, Ehd1*, and *Hmgcs2* were significantly up-regulated in HFpEF by 29, 8.34, and 3.27 times, respectively.

**Table 1:** The top 20 differently expressed m6A methylation peaks based on *P*-value.

**mRNA**	**Chromosome**	**Peak region**	**Lg (*P*-value)**	**log_**2**_ (fold-change)**	**Up/down**
Hspa1a	Chr17	3′UTR	−9.8	−3.95	Down
Ehd1	Chr19	3′UTR	−8.04	3.06	Up
Hmgcs2	Chr3	5′UTR	−7.66	1.71	Up
Fzd4	Chr7	Exon	−6.78	1.71	Up
Fbxl22	Chr9	Exon	−6.67	−1.82	Down
1810013L24Rik	Chr16	3′UTR	−5.71	1.99	Up
Adamts1	Chr16	3′UTR	−5.69	1.86	Up
Kdm3b	Chr18	Exon	−5.57	1.86	Up
Snapin	Chr3	Exon	−5.45	2.48	Up
Kank2	Chr9	3′UTR	−5.44	2.44	Up
Acot1	Chr12	Exon	−5.4	3.04	Up
Sdha	Chr13	3′UTR	−5.39	−0.611	Down
Lhfp	Chr3	5′UTR	−5.27	2.02	Up
Cfh	Chr1	Exon	−5.19	0.946	Up
Ywhae	Chr11	5′UTR	−5.01	1.4	Up
Fem1a	Chr17	3′UTR	−5.01	1.13	Up
Jun	Chr4	Exon	−4.91	−1.82	Down
Lbh	Chr17	3′UTR	−4.86	1.7	Up
Lmcd1	Chr6	3′UTR	−4.84	1.94	Up
Tmed7	Chr18	3′UTR	−4.77	1.51	Up

### Functional Annotation of the m6A Methylation by GO and KEGG Analyses

**Figure 5:**
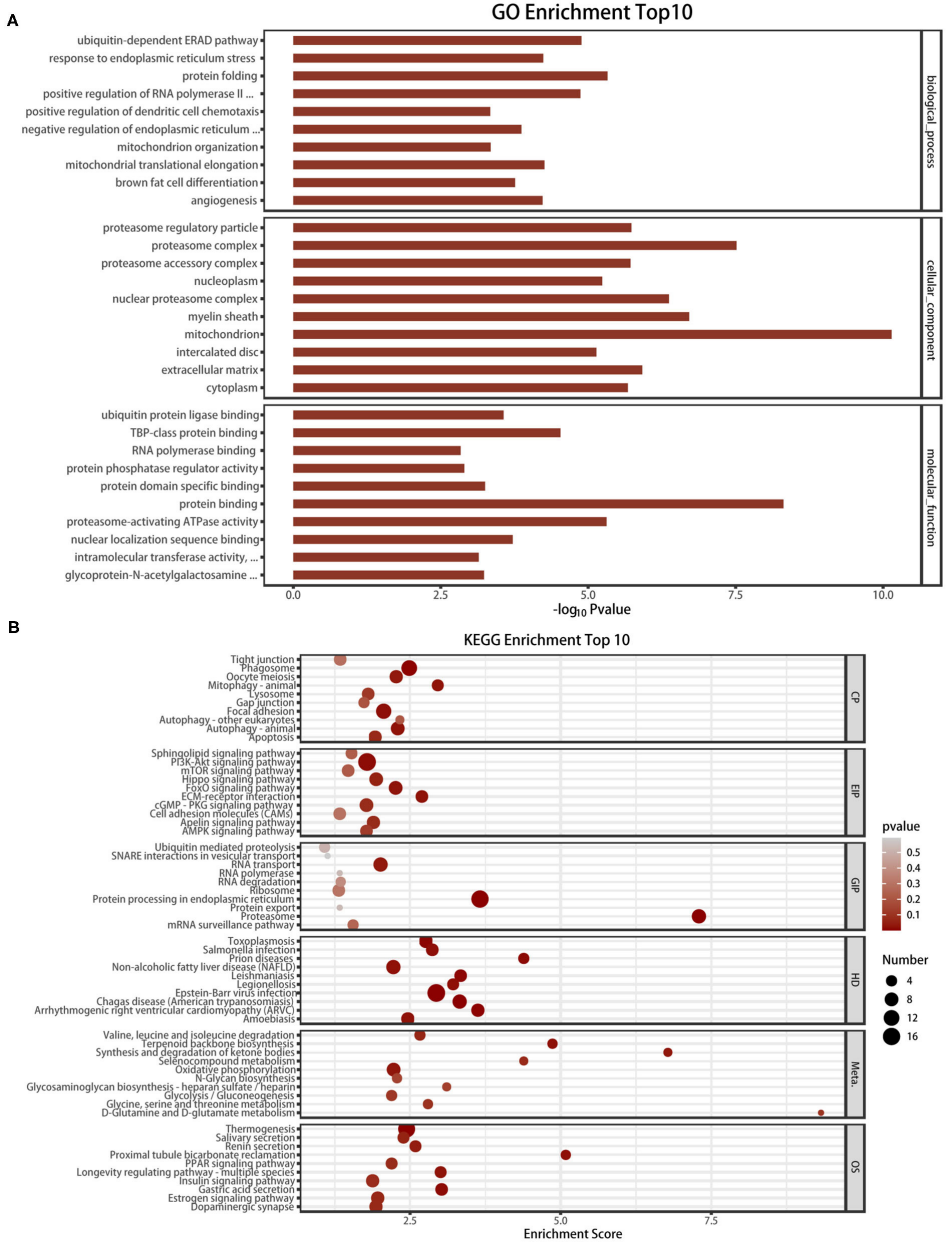
GO analysis and KEGG pathway analysis of the altered mRNA m6A modification. **(A)** The top 10 enriched GO terms of the m6A peaks. **(B)** The top 10 enriched pathways of m6A peaks.

reveal the role of m6A modification in HFpEF, the mRNAs with significantly altered m6A methylation level were subjected to gene functional annotation by the GO and KEGG pathway analyses. GO analysis was divided into three parts: biological process, cellular component, and molecular function (Figure 5A). Protein folding, ubiquitin-dependent ERAD pathway, and positive regulation of RNA polymerase II were the three most significantly enriched in biological process. Mitochondrion, proteasome complex, and myelin sheath were the three most significantly enriched in cellular component. Furthermore, protein binding, proteasome-activating ATPase activity, and TBP-class protein binding were the three most significantly enriched in molecular function. Through KEGG pathway analysis, we annotated the mRNAs with significantly altered m6A modification levels. These mRNAs were mostly enriched in the pathways including proteasome, protein processing in the endoplasmic reticulum, and PI3K-Akt signaling pathway (Figure 5B).

### Combined Analysis of m6A Modification and Gene Expression in HFpEF

**Figure 6:**
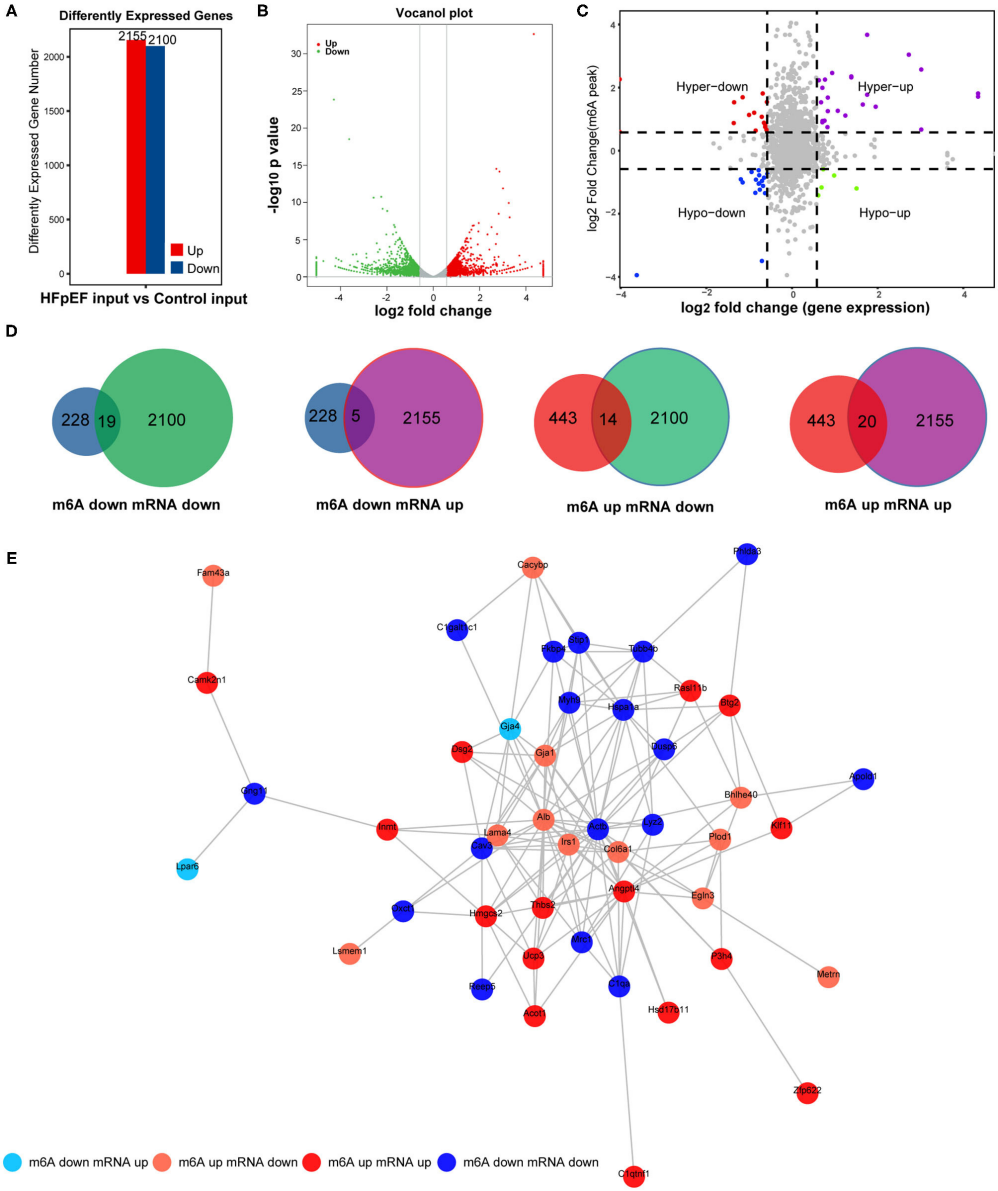
The combined analysis with MeRIP-seq and RNA-seq. **(A)** The up-regulated and down-regulated mRNAs by RNA-seq in HFpEF mice and control mice. **(B)** The volcano plot of differently expressed genes. **(C,D)** Four quadrant graph and Venn diagram show the correlation between mRNA m6A methylation and its mRNA expression. **(E)** The protein–protein interaction network shows the connection between the proteins of the genes used in the combined analysis.

To further demonstrate the association between m6A modification and gene expression, the level of mRNA alteration was high-throughput detected in HFpEF mice and control mice by RNA sequencing data of input experiments. A total of 4,255 differently expressed genes were identified (*P* < 0.05, log_2_ FC >1), in which 2,155 genes were significantly up-regulated and 2,100 genes were significantly down-regulated in HFpEF (Figure 6A), compared with control. The top 20 significantly altered mRNAs are presented in Table 2. The volcano plot (Figure 6B) shows the significantly up-regulated and down-regulated mRNAs between two groups.

**Table 2:** The top 20 differently expressed mRNAs based on *P*-value.

**mRNA**	***P*-value**	**log_**2**_ (fold-change)**	**Up/down**
Hmgcs2	2.25E−33	4.337564	Up
Hspa1b	1.45E−24	−4.29178	Down
Hspa1a	3.04E−19	−3.63182	Down
Acot1	2.95E−15	2.72059	Up
Spock2	6.94E−15	2.858137	Up
Angptl4	1.26E−12	3.011051	Up
Hsph1	1.70E−11	−2.2474	Down
Itgb6	2.26E−11	−2.57659	Down
Tcf23	1.16E−10	3.257808	Up
Cpxm2	6.96E−10	−2.18919	Down
Bdh1	1.39E−09	−1.98944	Down
Pth1r	3.28E−09	2.82245	Up
Myh7	1.00E−08	3.295993	Up
Scd4	5.83E−08	1.962302	Up
Col3a1	1.06E−07	−1.69663	Down
Hsd17b11	1.22E−07	1.747722	Up
Fmo2	1.35E−07	1.698478	Up
Serpinh1	1.98E−07	−1.6577	Down
Dusp4	2.06E−07	2.513462	Up
Thbs2	4.58E−07	1.646042	Up

Combined analysis of mRNA m6A modification and gene expression levels used peaks with log_2_ FC >0.58, *P* < 0.01 and the mRNA with log_2_ FC >1, *P* < 0.05. The association of m6A methylation and gene expression is presented in the quadrant graph (Figure 6C) and Venn diagram (Figure 6D). As shown, there were 58 mRNAs where both their m6A peaks and mRNA levels were altered significantly, among which the levels of 19 mRNAs were both down-regulated and the levels of 20 mRNAs were both up-regulated. Besides, there were five genes with down-regulated m6A peaks and up-regulated mRNA expression and 14 genes with up-regulated m6A peaks and down-regulated mRNA expression (Figures 6C,D). Furthermore, the protein–protein interaction network was performed to exhibit the junction between the proteins encoded by these identified genes (Figure 6E).

## Discussion

To the best of our knowledge, this is the first study to detect the m6A regulators in peripheral blood in cardiovascular diseases and the first study to explore the role of m6A methylation in HFpEF. Combined with the clinical case–control study and animal experiment, we showed the different expression patterns of m6A regulators in HFpEF patients and healthy controls and their association with the risk of HFpEF. Through MeRIP-seq, we obtained an m6A methylation panorama in a “two-hit” mouse model of HFpEF, which extended our knowledge of the critical role of m6A modification in HFpEF epigenetics.

Because it is difficult to obtain heart tissue samples from HFpEF patients, we detected the m6A regulators in PBMCs, although m6A RNA methylation in PBMCs may not reflect the post-transcriptional situation in the gene expression related to the function of the myocardium. The expression pattern of m6A regulators in the hearts of HFpEF mice is different from that in PBMCs of HFpEF patients, in which *FTO* and *YTHDC1* are up-regulated and *METTL3* is down-regulated. This may be explained by different sources of tissues. The altered expression pattern of m6A regulators in PBMCs of HFpEF patients represents the diagnostic potential of m6A regulators; however, the changed m6A regulators in heart tissue give inspiration to the pathological mechanisms and treatments.

Compared with the healthy controls, HFpEF showed higher expression of *METTL3, METTL4, KIAA1429, FTO*, and *YTHDF2* in peripheral blood (Figure 1). Similarly, it is reported that *METTL3, FTO, METTL14*, and *WTAP* are up-regulated in diabetes patients (34). Previous study reveals that the mRNA expression of *FTO* is positively correlated with glucose in diabetes patients (34); however, there is no correlation of m6A regulators with fasting glucose in our study. However, the correlation of m6A regulators with blood lipids is revealed. *METTL4* was negatively correlated with TC and HDL-C, and *KIAA1429* was negatively correlated with TC and LDL-C. Due to the cardioprotective function of HDL and the opposite roles of TC and LDL-C, KIAA1429 may play a cardioprotective role in HFpEF, but the role of METTL4 may be more complex. Interesting, *FTO* is up-regulated in both peripheral blood of HFpEF patients and hearts of HFpEF mice. As the core of the m6A methyltransferases, METTL3 can form a complex with METTL14 and WTAP to catalyze m6A modification on RNA (35). In contrast, a demethylase FTO could mediate the reversion of m6A methylation of RNA (36). Due to the alteration of m6A regulators in HFpEF, m6A modification resulted in both up-regulated peaks (433) and down-regulated peaks (228) in HFpEF. Recent studies have revealed that the expression pattern of m6A regulators and global level of m6A were changed in myocardial infarction, ischemia–reperfusion injury, myocardial hypertrophy, and HFrEF (16–19). METTL3 is increased in ischemia–reperfusion injury, but METTL14 was not significantly changed (18). Furthermore, FTO is decreased in HFrEF (16). The difference expression of m6A methyltransferases and demethylases in different cardiovascular diseases might be caused by physiopathologic differences or different organ sources.

By high-throughput measure of the m6A modification by MeRIP-seq, we found that the m6A modification levels of several mRNAs (i.e., *Alb, Ehd1, Hmgcs2*) related to the pathophysiological processes of HFpEF were significantly altered. The level of albumin (Alb) is an important hallmark of nutritional state, and a low serum Alb has been demonstrated to be a marker of myocardial fibrosis and exacerbates the prognosis of HFpEF (37, 38). Furthermore, we found that the m6A methylation of Alb was up-regulated in HFpEF and the mRNA of Alb was down-regulated. Ehd1 (Eps15 homology domain-containing protein 1) is recently identified as a novel interactor of Cx43 in the heart and plays a critical role in the pathological remodeling of gap junctions (39). Hmgcs2 is a ketone metabolic enzyme, and the level of Hmgcs2 in patients with arrhythmogenic cardiomyopathy is elevated, which leads to elevated plasma beta-hydroxybutyrate (β-OHB) and predicts major adverse cardiovascular events (MACE) (40). The altered m6A peaks in HFpEF have been associated with several protein processing by GO enrichment and KEGG pathway analyses, such as protein folding, ubiquitin, and protein binding, which means that the dysfunctional m6A methylation of the protein process plays a vital role in the development of HFpEF. Zhang et al. proved that activating the proteolytic function of the ubiquitin–proteasome system improves mouse survival in HFpEF (41). However, the results of the GO analysis and KEGG pathway analysis were not confirmed by phenotypical study, and we suggested using the METTL3 conditional knockout mouse model or FTO inhibitor to further verify the role of m6A in HFpEF. In addition, further research studies could be performed to confirm the exact protein level of these m6A methylated mRNAs.

Recent studies have revealed that m6A methylation induces the dysfunction of mRNA half-life and leads to mRNA instability (42). To better understand the mechanisms of m6A modification in HFpEF, we screened all the altered m6A peaks combined with the differentially expressed mRNAs. Consequently, 58 mRNAs were identified, whose m6A peak and gene level were both altered significantly, which could be divided into four parts: mRNA and m6A peaks both down-regulated (19), mRNA and m6A peaks both up-regulated (20), the m6A peak down-regulated and mRNA up-regulated (5), and the m6A peak up-regulated and mRNA down-regulated (14). The differently expressed level of m6A methylation will be recognized by “reader” protein and then induces different outcomes, for example, mRNA decay, mRNA stability, and mRNA translation (43). The m6A “reader” protein YTHDF2 is identified to control the half-life of target transcripts by mediating mRNA degradation, whereas YTHDF1 promotes translational effect.

In conclusion, to the best of our knowledge, this is the first study to explore the role of m6A methylation in HFpEF. Our study shows that the expression pattern of m6A regulators is changed in HFpEF. By MeRIP-seq, 661 m6A peaks were identified to be significantly altered in HFpEF mice, compared with control mice. The further combined analysis of m6A peaks and genes expression disclosed that there were 58 mRNAs significantly altered in HFpEF. These identified genes may be the critical regulators to interfere in the epigenetic regulation of HFpEF, and further exploring the fine regulation mechanism of m6A could open up a way to effective treatment for HFpEF.

### Limitations

Firstly, the sample size is small in this study, and we will further expand the sample size and explore the association of m6A regulators with the prognosis of HFpEF in the future. Secondly, the precise mechanism of m6A regulators in HFpEF needs to be studied in the future, for example, by using conditional knockout mouse model. Thirdly, m6A RNA methylation in PBMCs may not reflect the post-transcriptional situation in the gene expression related to the function of the myocardium.

## Data Availability Statement

The datasets presented in this study can be found in online repositories. The names of the repository/repositories and accession number(s) can be found at: https://www.ncbi.nlm.nih.gov/, PRJNA691715; https://www.ncbi.nlm.nih.gov/, PRJNA691685.

## Ethics Statement

The studies involving human participants were reviewed and approved by Ethics Committee at Zhongshan Hospital, Fudan University, China. The patients/participants provided their written informed consent to participate in this study. The animal study was reviewed and approved by the Animal Ethics Committee at Zhongshan Hospital, Fudan University, China.

## Author Contributions

BZ, AS, and JG were responsible for the design of the study and the writing the manuscript. YX and XC were responsible for data analysis work. HJ, WL, XW, YW, and YZ were responsible for the edit of the manuscript. All authors read and approved the final manuscript.

## Conflict of Interest

The authors declare that the research was conducted in the absence of any commercial or financial relationships that could be construed as a potential conflict of interest.
