# Supplementary material for: Alteration of m6A RNA Methylation in Heart Failure With Preserved Ejection Fraction
Source: Front Cardiovasc Med. 2021 Mar 5;8:647806. doi: 10.3389/fcvm.2021.647806 (PMC7973040; doi:10.3389/fcvm.2021.647806)
Supplement: Supplementary file 1 [file Table_1.DOCX]

**Table S1.** **Primers used in this study.**

| **Genes** | **Primer types** | **Primer Sequences （5’-3’）** |
| --- | --- | --- |
| Human |  |  |
| *METTL3* | Forward | TTGTCTCCAACCTTCCGTAGT |
| *METTL3* | Reverse | CCAGATCAGAGAGGTGGTGTAG |
| *METTL14* | Forward | AGTGCCGACAGCATTGGTG |
| *METTL14* | Reverse | GGAGCAGAGGTATCATAGGAAGC |
| *METTL4* | Forward | TCTGTGGTACACCAGTTGTCA |
| *METTL4* | Reverse | CCTTTTTACGGCAACAAGGTTCA |
| *WTAP* | Forward | CTTCCCAAGAAGGTTCGATTGA |
| *WTAP* | Reverse | TCAGACTCTCTTAGGCCAGTTAC |
| *KIAA1429* | Forward | AAGTGCCCCTGTTTTCGATAG |
| *KIAA1429* | Reverse | ACCAGACCATCAGTATTCACCT |
| *FTO* | Forward | ACTTGGCTCCCTTATCTGACC |
| *FTO* | Reverse | TGTGCAGTGTGAGAAAGGCTT |
| *ALKBH5* | Forward | CGGCGAAGGCTACACTTACG |
| *ALKBH5* | Reverse | CCACCAGCTTTTGGATCACCA |
| *YTHDF1* | Forward | ACCTGTCCAGCTATTACCCG |
| *YTHDF1* | Reverse | TGGTGAGGTATGGAATCGGAG |
| *YTHDF2* | Forward | AGCCCCACTTCCTACCAGATG |
| *YTHDF2* | Reverse | TGAGAACTGTTATTTCCCCATGC |
| *YTHDF3* | Forward | TCAGAGTAACAGCTATCCACCA |
| *YTHDF3* | Reverse | GGTTGTCAGATATGGCATAGGCT |
| *YTHDC1* | Forward | AACTGGTTTCTAAGCCACTGAGC |
| *YTHDC1* | Reverse | GGAGGCACTACTTGATAGACGA |
| *YTHDC2* | Forward | AGGACATTCGCATTGATGAGG |
| *YTHDC2* | Reverse | CTCTGGTCCCCGTATCGGA |
| *18S* | Forward | CGGCTACCACATCCAAGGAA |
| *18S* | Reverse | CCTGTATTGTTATTTTTCGTCACTACCT |
| Mouse |  |  |
| *METTL3* | Forward | CTGGGCACTTGGATTTAAGGAA |
| *METTL3* | Reverse | TGAGAGGTGGTGTAGCAACTT |
| *METTL14* | Forward | CTGAGAGTGCGGATAGCATTG |
| *METTL14* | Reverse | GAGCAGATGTATCATAGGAAGCC |
| *METTL4* | Forward | TGGGTGGTTACTGGATCATCT |
| *METTL4* | Reverse | AGCAAAGCACATAGCAGGAGC |
| *WTAP* | Forward | TAGACCCAGCGATCAACTTGT |
| *WTAP* | Reverse | CCTGTTTGGCTATCAGGCGTA |
| *KIAA1429* | Forward | ATAGATGTGGTTCGTTTTCCGTG |
| *KIAA1429* | Reverse | CACTATGGGCTCGTACTCCC |
| *FTO* | Forward | TTCATGCTGGATGACCTCAATG |
| *FTO* | Reverse | GCCAACTGACAGCGTTCTAAG |
| *ALKBH5* | Forward | GCATACGGCCTCAGGACATTA |
| *ALKBH5* | Reverse | TTCCAATCGCGGTGCATCTAA |
| *YTHDF1* | Forward | ACAGTTACCCCTCGATGAGTG |
| *YTHDF1* | Reverse | GGTAGTGAGATACGGGATGGGA |
| *YTHDF2* | Forward | GAGCAGAGACCAAAAGGTCAAG |
| *YTHDF2* | Reverse | CTGTGGGCTCAAGTAAGGTTC |
| *YTHDF3* | Forward | GATCAGCCTATGCCATATCTGAC |
| *YTHDF3* | Reverse | CCCCTGGTTGACTAAAAACACC |
| *YTHDC1* | Forward | GTCCACATTGCCTGTAAATGAGA |
| *YTHDC1* | Reverse | GGAAGCACCCAGTGTATAGGA |
| *YTHDC2* | Forward | GAAGATCGCCGTCAACATCG |
| *YTHDC2* | Reverse | GCTCTTTCCGTACTGGTCAAA |
